# E-Bayesian and Bayesian Estimation for the Lomax Distribution under Weighted Composite LINEX Loss Function

**DOI:** 10.1155/2021/2101972

**Published:** 2021-12-11

**Authors:** Afrah Al-Bossly

**Affiliations:** Department of Mathematics, College of Science and Humanities in Al-Kharj, Prince Sattam Bin Abdulaziz University, Al-Kharj 11942, Saudi Arabia

## Abstract

The main contribution of this work is the development of a compound LINEX loss function (CLLF) to estimate the shape parameter of the Lomax distribution (LD). The weights are merged into the CLLF to generate a new loss function called the weighted compound LINEX loss function (WCLLF). Then, the WCLLF is used to estimate the LD shape parameter through Bayesian and expected Bayesian (E-Bayesian) estimation. Subsequently, we discuss six different types of loss functions, including square error loss function (SELF), LINEX loss function (LLF), asymmetric loss function (ASLF), entropy loss function (ENLF), CLLF, and WCLLF. In addition, in order to check the performance of the proposed loss function, the Bayesian estimator of WCLLF and the E-Bayesian estimator of WCLLF are used, by performing Monte Carlo simulations. The Bayesian and expected Bayesian by using the proposed loss function is compared with other methods, including maximum likelihood estimation (MLE) and Bayesian and E-Bayesian estimators under different loss functions. The simulation results show that the Bayes estimator according to WCLLF and the E-Bayesian estimator according to WCLLF proposed in this work have the best performance in estimating the shape parameters based on the least mean averaged squared error.

## 1. Introduction

The expected Bayesian estimator is a new criterion for estimating the parameters, reliability and hazard functions, which consist of obtaining the expectation of Bayesian estimates with respect to the distributions of hyperparameters [1]. Monte Carlo simulation is used to compare the E-Bayesian estimator with the associated Bayesian estimator in terms of mean averaged squared error (MASE) [2, 3]. The E-Bayesian estimation method is efficient and easy to implement on real data [4]. Monte Carlo simulation is also used to compare new methods with corresponding Bayesian and maximum likelihood techniques [5]. The E-Bayesian method is used to obtain the likelihood function of the LD in the right-censored data type II and the parameter estimators of the LD in the right-censored data type II [6]. A new method is developed, to estimate failure probability which is defined based on formulas of the E-Bayesian estimate of the failure probability by [7]. The estimation under the LLF has a smaller deviation than the loss of the square error [8]. The E-Bayesian estimators are attained and built on the balanced squared error loss function by the gamma distribution as a conjugate solution prior for the indefinite scale parameter also using three diverse distributions for the hyperparameters [9]. E-Bayesian and hierarchical Bayesian estimation methods are used for estimating the scale parameter and reversed hazard rate of inverse Rayleigh distribution. These estimators are derived under squared error, entropy, and prophylactic loss functions [10]. The main purpose of this study is to develop a CLLF and use Bayesian and E-Bayesian estimators to estimate the shape parameters of the LD. Then, it will compare the proposed estimator with other methods including, maximum likelihood estimation (MLE), and Bayesian and E-Bayesian estimators under SELF, AS LF, ENLF, and CLLF.

LD is a widely used statistical model in reliability and life test research, especially in analyzing the data of life-testing experiments in engineering sciences, queuing theory, medicine, and physics. The probability density function (P.D.F) is

(1) $$\begin{array}{c} f\left(x;\beta ,\sigma \right)=\left\{\begin{array}{cc} \frac{\beta }{\sigma }{\left(1+\frac{x}{\sigma }\right)}^{-\left(\beta +1\right)}, & x\geq 0;\beta ,\sigma >0,\\ 0, & \text{o}.\text{w}. \end{array}\right. \end{array}$$

Hence, the C.D.F. is

(2) $$\begin{array}{c} F\left(x;\beta ,\sigma \right)=1-{\left(1+\frac{x}{\sigma }\right)}^{-\beta },\;x\geq 0, \end{array}$$

where *σ* > 0 is a scale parameter and *β* > 0 is a shape parameter. Also, the reliability function *R*(*t*) for the LD has been specified as follows:

(3) $$\begin{array}{c} R\left(t\right)={\left(1+\frac{t}{\sigma }\right)}^{-\beta },\;t\geq 0. \end{array}$$

## 2. Maximum Likelihood Estimation (MLE)

Suppose that $\underline{x}=\left(x_{1},x_{2},\ldots ,x_{n}\right)$, distributed according to the LD, is defined in (1). The likelihood of *β* can be described as

(4) $$\begin{array}{c} L\left(\underline{x}|\beta \right)=\prod_{i=1}^{n}\frac{\beta }{\sigma }{\left(1+\frac{x}{\sigma }\right)}^{-\left(\beta +1\right)}={\left(\frac{\beta }{\sigma }\right)}^{n}\exp\left[-\gamma \left(\beta +1\right)\right], \end{array}$$

where *γ*=∑_*i*=1_^*n*^ln(1+(*x*_*i*_/*σ*)) The logarithm of likelihood (4) is

(5) $$\begin{array}{c} \ln\;L\left(\beta ,\sigma \right)=n\;\ln\;\beta -n\;\ln\;\sigma -\left(\beta +1\right)\sum_{i=1}^{n}\ln\left(1+\frac{x_{i}}{\sigma }\right). \end{array}$$

As the parameter *σ* is assumed to be known, the MLE estimator of *β* is obtained by solving the equation

(6) $$\begin{array}{c} \frac{\partial \;\ln L\left(\beta ,\sigma \right)}{\partial \beta }=0,\\ \Rightarrow \frac{n}{\beta }-\sum_{i=1}^{n}\ln\left(1+\frac{x_{i}}{\sigma }\right)=0. \end{array}$$

Thus, the maximum likelihood estimates (MLEs) ${\hat{\beta }}_{\text{MLE}}$ of *β* is given by

(7) $$\begin{array}{c} {\hat{\beta }}_{\text{MLE}}=\frac{n}{\sum_{i=1}^{n}\ln\left(1+\left(x_{i}/\sigma \right)\right)}. \end{array}$$

## 3. Loss Functions

The Bayes estimation of a parameter *β* is based in minimization of a Bayesian loss (risk) function; $L\left(\hat{\beta },\beta \right)$ is defined as an average cost-of-error function:

(8) $$\begin{array}{c} \text{Risk}\left(\hat{\beta }\right)=E_{\beta }\left[L\left(\hat{\beta },\beta \right)\right]=\int_{\forall \beta }L\left(\hat{\beta },\beta \right)h\;\left(\beta |\underline{x}\right)\text{d}\beta . \end{array}$$

### 3.1. Squared Error Loss Function (SELF)

The SELF can be written as [11]

(9) $$\begin{array}{c} L\left(\hat{\beta },\beta \right)={\left(\hat{\beta }-\beta \right)}^{2}. \end{array}$$

The Bayes estimator of *β* with this loss function, denoted by ${\hat{\beta }}_{\text{BSE}}$, can be obtained as

(10) $$\begin{array}{c} {\hat{\beta }}_{\text{BSE}}=E_{h}\left(\beta |\underline{x}\right). \end{array}$$

### 3.2. LINEX Loss Function (LLF)

The LLF can be expressed as [12, 13]

(11) $$\begin{array}{c} L\left(\hat{\beta },\beta \right)\propto \left[\exp\left[c\left(\hat{\beta }-\beta \right)\right]-c\left(\hat{\beta }-\beta \right)-1\right],\;c\neq 0. \end{array}$$

The Bayes estimator of *β*, based on LLF and denoted by ${\hat{\beta }}_{\text{BL}}$, is given by

(12) $$\begin{array}{c} {\hat{\beta }}_{\text{BL}}=-\frac{1}{c}\text{Ln}\left[E_{\beta }\exp\left[-c\beta \right]\right],\;c\neq 0, \end{array}$$

provided that *E*_*β*_=(*e*^−*cβ*^) exists and is finite.

### 3.3. Asymmetric Loss Function (ASLF)

Asymmetric loss function is defined as [14]

(13) $$\begin{array}{c} L\left(\hat{\beta },\beta \right)={\left(\sqrt{\frac{\beta }{\hat{\beta }}}-\sqrt{\frac{\hat{\beta }}{\beta }}\right)}^{2}=\frac{\beta }{\hat{\beta }}-\frac{\hat{\beta }}{\beta }-2. \end{array}$$

The Bayes estimator of *β*, based on ASLF and denoted by ${\hat{\beta }}_{\text{BAS}}$, is given by

(14) $$\begin{array}{c} {\hat{\beta }}_{\text{BAS}}={\left(\frac{E\left(\beta^{-1}|\underline{x}\right)}{E\left(\beta |\underline{x}\right)}\right)}^{-\left(1/2\right)}. \end{array}$$

### 3.4. Entropy Loss Function (ANLF)

The ENLF for *β* can be expressed as [15]

(15) $$\begin{array}{c} L\left(\hat{\beta },\beta \right)\propto \left(\frac{\hat{\beta }}{\beta }\right)-\ln\left(\frac{\hat{\beta }}{\beta }\right)-1. \end{array}$$

The Bayes estimator of *β*, denoted by ${\hat{\beta }}_{\text{BEN}}$ is the value $\hat{\beta }$ which minimizes equation (15) and is given as

(16) $$\begin{array}{c} {\hat{\beta }}_{\text{BEN}}={\left[E_{\beta }\left(\beta^{-1}\right)\right]}^{-1}, \end{array}$$

provided that *E*_*β*_(*β*^−1^) exists and is finite.

### 3.5. Composite LINEX Loss Function (CLLF)

CLLF was introduced by Zhang [16] as follows:

(17) $$\begin{array}{c} L\left(\hat{\beta },\beta \right)=L_{c}\;\left(\hat{\beta },\beta \right)+L_{-c}\left(\hat{\beta },\beta \right)=\exp\left[-c\left(\hat{\beta },\beta \right)\right]+\exp\left[c\left(\hat{\beta },\beta \right)\right]-2,\;c>0. \end{array}$$

The Bayes estimator of *β*, denoted by ${\hat{\beta }}_{\text{BCL}}$, is given by

(18) $$\begin{array}{c} {\hat{\beta }}_{\text{BCL}}=\frac{1}{2c}\ln\left(\frac{E\left(\exp\left[c\beta \right]|\underline{x}\right)}{E\left(\exp\left[-c\beta \right]|\underline{x}\right)}\right). \end{array}$$

### 3.6. Weighted Composite LINEX Loss Function

The researcher proposes this loss function depending on weighting CLLF as follows:

(19) $$\begin{array}{c} L_{w}\left(\hat{\beta },\beta \right)=w\;\left(\beta \right)L\left(\hat{\beta },\beta \right)=w\left(\beta \right)L_{c}\left(\hat{\beta },\beta \right)+w\left(\beta \right)L_{-c}\left(\hat{\beta },\beta \right)\\ =w\left(\beta \right)\exp\left[-c\left(\hat{\beta },\beta \right)\right]+w\left(\beta \right)\exp\left[c\left(\hat{\beta },\beta \right)\right]-2,\\ c>0, \end{array}$$

where *w*(*β*) represents the proposed weighted function, which is given by

(20) $$\begin{array}{c} w\left(\beta \right)=\exp\left[-\omega \beta \right]. \end{array}$$

According to the abovementioned loss function, we drive the corresponding Bayes estimators for *β* using Risk function $R\left(\hat{\beta }-\beta \right)$, which minimizes the posterior risk:

(21) $$\begin{array}{c} R\left(\hat{\beta }-\beta \right)=E\left[L_{w}\left(\hat{\beta },\beta \right)\right]=\int_{0}^{\infty }\left(w\left(\beta \right)\left[\exp\left[-c\left(\hat{\beta }-\beta \right)\right]+\exp\left[c\left(\hat{\beta }-\beta \right)\right]-2\right]\right)h\left(\beta |\underline{x}\right)\text{d}\beta \\ =\left\{\begin{array}{c} \int_{0}^{\infty }\exp\left[-\omega \beta \right]\exp\left[-c\left(\hat{\beta }-\beta \right)\right]h\left(\beta |\underline{x}\right)\text{d}\beta \\ +\int_{0}^{\infty }\exp\left[-\omega \beta \right]\exp\left[c\left(\hat{\beta }-\beta \right)\right]h\left(\beta |\underline{x}\right)\text{d}\beta -2\int_{0}^{\infty }\exp\left[-\omega \beta \right]h\left(\beta |\underline{x}\right)\text{d}\beta \end{array}\right\}\\ =\left\{\begin{array}{c} \exp\left[-c\hat{\beta }\right]\int_{0}^{\infty }\exp\left[-\beta \left(\omega -c\right)\right]h\left(\beta |\underline{x}\right)\text{d}\beta \\ \;+\exp\left[c\hat{\beta }\right]\int_{0}^{\infty }\exp\left[-\beta \left(\omega +c\right)\right]h\left(\beta |\underline{x}\right)\text{d}\beta -2\int_{0}^{\infty }\exp\left[-\omega \right]h\left(\beta |\underline{x}\right)\text{d}\beta \; \end{array}\right\}\\ =\left\{\begin{array}{c} \exp\left[-c\hat{\beta }\right]E_{\beta }\left[\left(\exp\left[-\beta \left(\omega -c\right)\right]|\underline{x}\right)\right]\;\\ \;+\exp\left[c\hat{\beta }\right]\;E_{\beta }\left[\left(\exp\left[-\beta \left(\omega +c\right)\right]|\underline{x}\right)\right]-2E_{\beta }\left[\exp\left[\left(-\omega \beta |\underline{x}\right)\right]\right] \end{array}\right\},\\ \frac{\partial R\left(\hat{\beta }-\beta \right)}{\partial \hat{\beta }}=-c\;\exp\left[-c\hat{\beta }\right]E_{\beta }\left[\left(\exp\left[-\beta \left(\omega -c\right)\right]|\underline{x}\right)\right]+c\;\exp\left[c\hat{\beta }\right]E_{\beta }\left[\left(\exp\left[-\beta \left(\omega +c\right)\right]|\underline{x}\right)\right],\\ \text{By letting}\frac{\partial R\left(\hat{\beta }-\beta \right)}{\partial \hat{\beta }}=0. \end{array}$$

The Bayes estimator for the parameter *β* under the WCLLF, denoted by ${\hat{\beta }}_{\text{WBCL}}$, is given by

(22) $$\begin{array}{c} {\hat{\beta }}_{\text{WBCL}}=\frac{1}{2c}\ln\left[\frac{E_{\beta }\left[\left(\exp\left[-\beta \left(\omega -c\right)\right]|\underline{x}\right)\right]}{E_{\beta }\left[\left(\exp\left[-\beta \left(\omega +c\right)\right]|\underline{x}\right)\right]}\right]. \end{array}$$

Note: composite CLLF is a special case of WCLLF when *ω*=0 in equation (8). It means the WCLLF is a generalizing of CLLF.

## 4. Bayesian Estimation

This section spotlights to derive Bayesian estimates of the shape parameter *β* of the LD. We use six different loss functions, including SELF, ASLF, ENLF, LLF, CLLF, and WCLLF. We use the gamma (*z*, *k*) as a conjugate prior of *β* and its density function as follows:

(23) $$\begin{array}{c} \pi \left(\beta |z,k\right)=\frac{k^{z}}{\Gamma \left(z\right)}\beta^{z-1}\exp\left[-k\beta \right],\;z,k>0,\beta >0. \end{array}$$

Based on equations (4) and (23), the posterior density function of *β* given as $\underline{x}$ is

(24) $$\begin{array}{c} h\left(\beta |\underline{x}\right)=\frac{L\left(\beta ,\underline{x}\right)\pi \left(\beta |z,k\right)}{\int_{0}^{\infty }L\left(\beta ,\underline{x}\right)\pi \left(\beta |z,k\right)\text{d}\beta }=\frac{{\left(\gamma +k\right)}^{n+z}\beta^{n+z-1}\exp\left[-\beta \left(\gamma +k\right)\right]}{\Gamma \left(n+z\right)}, \end{array}$$

where *γ*=∑_*i*=1_^*n*^ln(1+(*x*_*i*_/*σ*)).

### 4.1. Bayesian Estimation Based on SELF

The Bayesian estimator ${\hat{\beta }}_{\text{BSE}}$, of *β* with SELF, is defined as

(25) $$\begin{array}{c} {\hat{\beta }}_{\text{BSE}}=E\left(\beta |\underline{x}\right)=\int_{0}^{\infty }\beta h\left(\beta |\underline{x}\right)\text{d}\beta \\ =\int_{0}^{\infty }\frac{{\left(\gamma +k\right)}^{n+z}\beta^{n+a}\exp\left[-\beta \left(\gamma +k\right)\right]}{\Gamma \left(n+z\right)}\text{d}\beta \\ =\frac{n+z}{\gamma +k}. \end{array}$$

### 4.2. Bayesian Estimation Based on LLF

Based on LLF, we can give the Bayesian estimation, ${\hat{\beta }}_{\text{BL}}$, of *β* as

(26) $$\begin{array}{c} {\hat{\beta }}_{\text{BL}}=-\frac{1}{c}\text{Ln}\left[E_{\beta }\exp\left[-c\beta \right]\right]\\ =-\frac{1}{c}\text{Ln}\int_{0}^{\infty }\exp\left[-c\beta \right]h\left(\beta |\underline{x}\right)\beta ,\\ {\hat{\beta }}_{\text{BL}}=-\frac{1}{c}\text{Ln}\int_{0}^{\infty }\frac{{\left(\gamma +k\right)}^{n+z}\beta^{n+z-1}\exp\left[-\beta \left(\gamma +k+c\right)\right]}{\Gamma \left(n+z\right)}\text{d}\beta \\ =\frac{n+z}{c}\ln\left(1+\frac{c}{\gamma +k}\right). \end{array}$$

### 4.3. Bayesian Estimation Based on ASLF

Under ASLF, the Bayesian estimation, ${\hat{\beta }}_{\text{BS}}$, of *β* can be expressed as

(27) $$\begin{array}{c} {\hat{\beta }}_{\text{BS}}={\left(\frac{E\left(\beta^{-1}|\underline{x}\right)}{E\left(\beta |\underline{x}\right)}\right)}^{-\left(1/2\right)}=\frac{I_{1}}{I_{2}}, \end{array}$$

where

(28) $$\begin{array}{c} I_{1}=E\left(\beta^{-1}|\underline{x}\right)=\int_{0}^{\infty }\frac{{\left(\gamma +k\right)}^{n+z}\beta^{n+z-2}\exp\left[-\beta \left(\gamma +k\right)\right]}{\Gamma \left(n+z\right)}\text{d}\beta ={\left(\gamma +k\right)}^{n+z}\frac{\Gamma \left(n+z-1\right)}{{\left(\gamma +k\right)}^{n+z-1\;}},\\ I_{2}=E\left(\beta |\underline{x}\right)=\int_{0}^{\infty }\frac{{\left(\gamma +k\right)}^{n+z}\beta^{n+z}\exp\left[-\beta \left(\gamma +k\right)\right]}{\Gamma \left(n+z\right)}\text{d}\beta =\frac{n+z}{\gamma +k}, \end{array}$$

so

(29) $$\begin{array}{c} {\hat{\beta }}_{\text{BAS}}=\frac{\sqrt{\left(n+z-1\right)\left(n+z\right)}}{\gamma +k}. \end{array}$$

### 4.4. Bayesian Estimation-Based ENLF

Based on ENLF, the Bayesian estimation, ${\hat{\beta }}_{\text{BE}}$, of *β* can be shown to be

(30) $$\begin{array}{c} {\hat{\beta }}_{\text{BEN}}=E\left(\beta^{-1}|\underline{x}\right)\\ =\int_{0}^{\infty }\beta^{-1}\;h\left(\beta |\underline{x}\right)\text{d}\beta \\ ={\left[\int_{0}^{\infty }\frac{{\left(\gamma +k\right)}^{n+z}\beta^{n+z-2}\exp\left[-\beta \left(\gamma +k\right)\right]}{\Gamma \left(n+z\right)}\text{d}\beta \right]}^{-1}\\ =\frac{n+z-1}{\gamma +k}. \end{array}$$

### 4.5. Bayesian Estimation Based on CLLF

Based on CLLF, the Bayesian estimator, ${\hat{\beta }}_{\text{BCL}}$, of *β*, can be obtained as follows [16]:

(31) $$\begin{array}{c} {\hat{\beta }}_{\text{BCL}}=\frac{1}{2c}\ln\left(\frac{E_{\beta }\left(\exp\left[c\beta \right]|\underline{x}\right)}{E_{\beta }\left(\exp\left[-c\beta \right]|\underline{x}\right)}\right)=\frac{1}{2c}\ln\left(\frac{I_{3}}{I_{4}}\right), \end{array}$$

where

(32) $$\begin{array}{c} I_{3}=E_{\beta }\left(\exp\left[c\beta \right]|\underline{x}\right)=\int_{0}^{\infty }\exp\left[c\beta \right]\frac{{\left(\gamma +k\right)}^{n+z}}{\Gamma \left(n+a\right)}\beta^{n+z-1}\exp\left[-\beta \left(\gamma +k\right)\right]\text{d}\beta \\ =\frac{{\left(\gamma +k\right)}^{n+z}}{\Gamma \left(n+a\right)}\int_{0}^{\infty }\beta^{n+z-1}\exp\left[-\beta \left(\gamma +K-c\right)\right]\text{d}\beta \\ =\frac{{\left(\gamma +k\right)}^{n+z}}{{\left(\gamma +K-c\right)}^{n+z}},\\ I_{4}=E_{\beta }\left(\exp\left[-c\beta \right]|\underline{x}\right)=\int_{0}^{\infty }\exp\left[-c\beta \right]\frac{{\left(\gamma +k\right)}^{n+z}}{\Gamma \left(n+z\right)}\beta^{n+z-1}\exp\left[-\beta \left(\gamma +k\right)\right]\text{d}\beta \\ =\frac{{\left(\gamma +k\right)}^{n+z}}{\Gamma \left(n+z\right)}\int_{0}^{\infty }\beta^{n+z-1}\exp\left[-\beta \left(\gamma +K+c\right)\right]\text{d}\beta \\ =\frac{{\left(\gamma +k\right)}^{n+z}}{{\left(\gamma +K+c\right)}^{n+z}}, \end{array}$$

so

(33) $$\begin{array}{c} {\hat{\beta }}_{\text{BCL}}=\frac{1}{2c}\ln\left[\frac{{\left(\left(\gamma +k\right)/\left(\gamma +k-c\right)\right)}^{n+z}}{{\left(\left(\gamma +k\right)/\left(\gamma +k+c\right)\right)}^{n+z}}\right]\\ =\frac{n+z}{2c}\ln\left(\frac{\gamma +k+c}{\gamma +k-c}\right). \end{array}$$

### 4.6. Bayesian Estimation Based on WCLLF

Under the WCLLF, the Bayesian estimation, ${\hat{\beta }}_{\text{WBCL}}$, of *β*, can be shown as

(34) $$\begin{array}{c} {\hat{\beta }}_{\text{WBCL}}=\frac{1}{2c}\ln\left[\frac{E_{\beta }\left[\left(\exp\left[-\beta \left(\omega -c\right)\right]|\underline{x}\right)\right]}{E_{\beta }\left[\left(\exp\left[-\beta \left(\omega +c\right)\right]|\underline{x}\right)\right]}\right]=\frac{1}{2c}\ln\left(\frac{I_{5}}{I_{6}}\right), \end{array}$$

where

(35) $$\begin{array}{c} I_{5}=E_{\beta }\left[\left(\exp\left[-\beta \left(\omega -c\right)\right]|\underline{x}\right)\right]\\ =\int_{0}^{\infty }\exp\left[-\beta \left(\omega -c\right)\right]\frac{{\left(\gamma +k\right)}^{n+z}}{\Gamma \left(n+z\right)}\beta^{n+z-1}\exp\left[-\beta \left(\gamma +k\right)\right]\text{d}\beta \\ =\frac{{\left(\gamma +k\right)}^{n+z}}{\Gamma \left(n+z\right)}\int_{0}^{\infty }\beta^{n+z-1}\exp\left[-\beta \left(\gamma +K+\omega -c\right)\right]\text{d}\beta \\ =\frac{{\left(\gamma +k\right)}^{n+z}}{{\left(\gamma +K+\omega -c\right)}^{n+z}},\\ I_{6}=\;E_{\beta }\left[\left(\exp\left[-\beta \left(\omega +c\right)\right]|\underline{x}\right)\right]\\ =\int_{0}^{\infty }\exp\left[-\beta \left(\omega +c\right)\right]\frac{{\left(\gamma +k\right)}^{n+z}}{\Gamma \left(n+z\right)}\beta^{n+z-1}\exp\left[-\beta \left(\gamma +k\right)\right]\text{d}\beta \\ =\frac{{\left(\gamma +k\right)}^{n+z}}{\Gamma \left(n+z\right)}\int_{0}^{\infty }\beta^{n+z-1}\exp\left[-\beta \left(\gamma +K+\omega +c\right)\right]\text{d}\beta \\ =\frac{{\left(\gamma +k\right)}^{n+z}}{{\left(\gamma +K+\omega +c\right)}^{n+z}}. \end{array}$$

So,

(36) $$\begin{array}{c} {\hat{\beta }}_{\text{WBCL}}=\frac{1}{2c}\ln\left[\frac{{\left(\left(\gamma +k\right)/\left(\gamma +k+\omega -c\right)\right)}^{n+z}}{{\left(\left(\gamma +k\right)/\left(\gamma +k+\omega +c\right)\right)}^{n+z}}\right]\\ =\frac{n+z}{2c}\ln\left(\frac{\gamma +k+\omega +c}{\gamma +k+\omega -c}\right). \end{array}$$

## 5. E-Bayesian Estimation

In this section, we consider the E-Bayes estimates of the shape parameter *β* of the LD, by using six different loss functions, including SELF, ASLF, ENLF, LLF, CLLF, and WCLLF. Based on [17], the prior parameters *a* and *k* should be selected to guarantee that *π*(*β|a*, *k*) given in (23) is a decreasing function of *β*; the derivative of *π*(*β|z*, *k*) with respect to *β* is

(37) $$\begin{array}{c} \frac{\partial \pi \left(\beta |z,k\right)}{\partial \beta }=\frac{k^{z}}{\Gamma \left(z\right)}\beta^{z-2}\exp\left[-k\beta \right]\left(\beta -1-k\beta \right),\;0<z<1,k>0. \end{array}$$

Note that *z* > 0, *k* > 0, and *β* > 0; it follows 0 < *z* < 1 and *k* > 0 due to (∂*π*(*β|z*, *k*)/∂*β*) < 0, which equals to *z* − 1 − *kβ* < 0, and therefore, *π*(*β|z*, *k*) is a decreasing function of *β*. Assuming that *a* and *k* are independent with bivariate density function, *π*(*z*, *k*)=*π*_1_(*z*)*π*_2_(*k*) can be written as

(38) $$\begin{array}{c} {\hat{\beta }}_{\text{EB}}=E\left(\hat{\beta }|\underline{x}\right)=\int \int_{\forall \tau }{\hat{\beta }}_{B}\left(z,k\right)\pi \left(z,k|\underline{x}\right)\text{d}a\text{d}k, \end{array}$$

where ${\hat{\beta }}_{B}\left(z,k\right)$ is the Bayes estimator of *β* given by equations (25), (26), (29), (30), (33), and (36). We can choose uniform distribution as its prior distribution:

(39) $$\begin{array}{c} \pi \left(z,k|\underline{x}\right)=\frac{1}{v},\;0<z<1,0<k<v. \end{array}$$

### 5.1. E-Bayesian Estimation of Parameter *β* under SELF

For SELF, the E-Bayesian estimation, ${\hat{\beta }}_{\text{EBSE}}$, of *β* is obtained according to (25), (38), and (39) as

(40) $$\begin{array}{c} {\hat{\beta }}_{\text{EBSE}}=\int \int_{\forall \tau }{\hat{\beta }}_{\text{BSE}}\pi \left(z,k|\underline{x}\right)\text{d}a\text{d}k\\ =\int_{0}^{1}\int_{1}^{v}\frac{1}{v}\frac{n+z}{\gamma +k}\text{d}a\text{d}k\\ =\frac{1}{v}\int_{0}^{v}\frac{1}{T\gamma +k}\int_{0}^{1}\left(n+z\right)\text{d}z\text{d}k\\ =\frac{2n+1}{2v}\int_{0}^{v}\frac{1}{\gamma +k}\text{d}k\\ =\frac{2n+1}{2v}\int_{T}^{\gamma +k}\frac{1}{x}\text{d}x\\ =\frac{2n+1}{2v}\ln\left(\gamma +v\right)-\ln\left(\gamma \right). \end{array}$$

### 5.2. E-Bayesian Estimation under LLF

The E-Bayesian estimation, ${\hat{\beta }}_{\text{EBL}}$, of *β*, under the LLF and based on equations (26), (38), and (39), is given as follows:

(41) $$\begin{array}{c} {\hat{\beta }}_{\text{EBL}}=\int \int_{\forall \tau }{\hat{\beta }}_{\text{BL}}\pi \left(z,k|\underline{x}\right)\text{d}z\text{d}k\\ =\int_{0}^{1}\int_{1}^{v}\frac{1}{v}\frac{n+z}{c}\ln\left(\frac{\gamma +k+c}{\gamma +k}\right)\text{d}z\text{d}k\\ =\frac{1}{cv}\int_{0}^{v}\ln\left(\frac{\gamma +k+c}{\gamma +k}\right)\int_{0}^{1}\left(n+z\right)\text{d}z\text{d}k\\ =\frac{2n+1}{2cv}\int_{0}^{v}\ln\left(\frac{\gamma +k+c}{\gamma +k}\right)\text{d}k\\ =\frac{2n+1}{2cv}\int_{0}^{v}\ln\left(\gamma +k+c\right)-\ln\left(\gamma +k\right)\text{d}k\\ =\left\{\frac{2n+1}{2cv}\left[\;\left(\gamma +c+v\right)\ln\left(\gamma +c+v\right)-\left(\gamma +c\right)\ln\left(\gamma +c\right)-\left(\gamma +v\right)\ln\left(\gamma +v\right)+\left(\gamma \right)\ln\left(\gamma \right)\right]\right\}. \end{array}$$

### 5.3. E-Bayesian Estimation under an ASLF

Based on equations (29), (38), and (39), the E-Bayesian estimation, ${\hat{\beta }}_{\text{EBE}}$, of *β* under ASLF is [18]

(42) $$\begin{array}{c} {\hat{\beta }}_{\text{EBAS}}=\int \int_{\forall \tau }{\hat{\beta }}_{\text{EBS}}\pi \left(z,k|\underline{x}\right)\text{d}z\text{d}k\\ =\int_{0}^{1}\int_{0}^{v}\frac{1}{v}\frac{\sqrt{\left(n+z-1\right)\left(n+z\right)}}{\gamma +k}\text{d}z\text{d}k\\ =\frac{1}{v}\ln\frac{\gamma +v}{\gamma }\int_{0}^{1}\sqrt{\left(n+z-1\right)\left(n+z\right)}\text{d}z. \end{array}$$

### 5.4. E-Bayesian Estimation under ENLF

Based on equations (30), (38), and (39), the E-Bayesian estimation, ${\hat{\beta }}_{\text{EBE}}$, of *β* under ENLF is

(43) $$\begin{array}{c} {\hat{\beta }}_{\text{EBEN}}=\int \int_{\forall \tau }{\hat{\beta }}_{\text{EBE}}\pi \left(z|\underline{x}\right)\text{d}z\text{d}k\\ =\int_{0}^{1}\int_{0}^{v}\frac{1}{v}\frac{n+a-1}{\gamma +k}\text{d}z\text{d}k\\ =\frac{2n-1}{2v}\int_{0}^{v}\frac{1}{\gamma +k}\text{d}k\\ =\frac{2n-1}{2v}\int_{\gamma }^{\gamma +v}\frac{1}{x}\text{d}x\\ =\frac{2n-1}{2v}\ln\left(\gamma +v\right)-\ln\left(\gamma \right). \end{array}$$

### 5.5. E-Bayesian Estimation of Parameter *β* under CLLF

Based on equations (33), (38), and (39), the E-Bayesian estimation, ${\hat{\beta }}_{\text{EBCL}}$, of *β* under CLLF is [17]

(44) $$\begin{array}{c} {\hat{\beta }}_{\text{EBCL}}=\int \int_{\forall \tau }{\hat{\beta }}_{\text{EBCL}}\pi \left(z,k|\underline{x}\right)\text{d}z\text{d}k\\ =\int_{0}^{1}\int_{1}^{v}\frac{1}{v}\frac{n+z}{2c}\ln\left(\frac{\gamma +k+c}{\gamma +k-c}\right)\text{d}z\text{d}k\\ =\frac{1}{2cv}\int_{0}^{v}\ln\left(\frac{\gamma +k+c}{\gamma +k-c}\right)\int_{0}^{1}\left(n+z\right)\text{d}z\text{d}k\\ =\frac{2n+1}{4cv}\int_{0}^{v}\ln\left(\frac{T+k+c}{\gamma +k-c}\right)\text{d}k\\ =\frac{2n+1}{4cv}\int_{0}^{v}\ln\left(\gamma +k+c\right)-\ln\left(\gamma +k-c\right)\text{d}k\\ =\left\{\frac{2n+1}{4cv}\left[\left(\gamma +c+v\right)\ln\left(\gamma +c+v\right)-\left(\gamma +c\right)\ln\left(\gamma +c\right)-\left(\gamma -c+v\right)\ln\left(\gamma -c+v\right)+\left(\gamma -c\right)\ln\left(\gamma -c\right)\right]\right\}. \end{array}$$

### 5.6. E-Bayesian Estimation WCLLF

Based on proposed loss function WCLLF and according to equations (36), (38), and (39), we get the E-Bayesian estimation, ${\hat{\beta }}_{\text{EWBCL}}$, of *β* as follows:

(45) $$\begin{array}{c} {\hat{\beta }}_{\text{EWBCL}}=\int \int_{\forall \tau }{\hat{\beta }}_{\text{EWBCL}}\pi \left(z,k|\underline{x}\right)\text{d}z\text{d}k\\ =\int_{0}^{1}\int_{1}^{v}\frac{1}{v}\frac{n+z}{2c}\ln\left(\frac{\gamma +k+\omega +c}{\gamma +k+\omega -c}\right)\text{d}z\text{d}k\\ =\frac{1}{2cv}\int_{0}^{v}\ln\left(\frac{\gamma +k+\omega +c}{\gamma +k+\omega -c}\right)\int_{0}^{1}\left(n+z\right)\text{d}z\text{d}k\\ =\frac{2n+1}{4cv}\int_{0}^{v}\ln\left(\frac{\gamma +k+\omega +c}{T+k+\omega -c}\right)\text{d}k\\ =\frac{2n+1}{4cv}\int_{0}^{v}\ln\left(\gamma +k+\omega +c\right)-\ln\left(\gamma +k+\omega -c\right)\text{d}k\\ =\left\{\frac{2n+1}{4cv}\left[\left(\gamma +\omega +c+v\right)\ln\left(\gamma +\omega +c+v\right)-\left(\gamma +\omega +c\right)\ln\left(\gamma +\omega +c\right)-\left(\gamma +\omega -c+v\right)\ln\left(\gamma +\omega -c+v\right)+\left(\gamma +\omega -c\right)\ln\left(\gamma +\omega -c\right)\right]\right\}. \end{array}$$

## 6. Simulation and Results

In order to examine the performance of the estimators obtained in Sections 4 and 5, we used a Monte Carlo simulation study, according to the following steps:

(1) Select sample size *n*=25,50,75, and 100 with the parameter (*β*=1,1.5, and 2).
(2) Determine the value (*v*)=1, *c* = (0.5 and 1.5), (*a*, *k*)=(0.6, 0.5), and *ω*=0.5.
(3) For given sample size *n*, with known *σ*=3, generate *x*_1_, *x*_2_,…, *x*_*n*_ from *x*_*i*_=*σ*[(1 − *U*_*i*_)^−(1/*β*)^ − 1].
(4) MLE estimation, ${\hat{\beta }}_{\text{MLE}}$, of *β* is computed from equation (7).
(5) Bayesian estimation, ${\hat{\beta }}_{\text{BSE}}$, ${\hat{\beta }}_{\text{BL}}$, ${\hat{\beta }}_{\text{BS}}$, ${\hat{\beta }}_{\text{BE}}$, ${\hat{\beta }}_{\text{BCL}}$, and ${\hat{\beta }}_{\text{WBCL}}$, of *β* is computed from equations (25), (26), (29), (30), (33), and (36), respectively.
(6) E-Bayesian estimation, ${\hat{\beta }}_{\text{EBSE}}$, ${\hat{\beta }}_{\text{EBL}}$, ${\hat{\beta }}_{\text{EBS}}$, ${\hat{\beta }}_{\text{EBE}}$, ${\hat{\beta }}_{\text{EBCL}}$, and ${\hat{\beta }}_{\text{EWBCL}}$, of *β* is computed from equations (40)–(45), respectively.
(7) Steps 3 to 6 are repeated 10,000 times. We then compute the average estimates (AE) and the mean averaged squared error (MASE) for each estimate (say $\hat{\beta }$) was calculated by using
  (46) $$\begin{array}{c} \text{MASE}\left(\hat{\beta }\right)=\frac{1}{10000}\sum_{i=1}^{10000}{\left({\hat{\beta }}_{i}-\beta \right)}^{2},\\ \text{AE}\left(\hat{\beta }\right)=\frac{1}{10000}\sum_{i=1}^{10000}{\hat{\beta }}_{i}, \end{array}$$
where $\hat{\beta }$ is the estimate at the *i*^th^ run.
(8) The computational results are displayed in Tables 1–4.

From Tables 1–4, we have the following observations:

1. The estimated values of *β* is very close to the real values when the sample size increases for all cases; also, the differences between average estimates and the true value of the different estimates decrease as *n* increases
2. The E-Bayesian estimation of *β* with the proposed loss function WCLLF has the best estimate due to the smallest value of MASE's comparing with all other estimates
3. The Bayesian estimation of *β* under the proposed loss function WCLLF has the minimum MASE's comparing with all other Bayesian estimates
4. The E-Bayesian estimation of *β* with the proposed loss function WCLLF has the best estimate due to the smallest value of MASE's comparing with all other E-Bayesian estimates
5. E-Bayesian estimators perform better than the Bayesian estimator in terms of MASE, for all sample sizes *n* and all cases
6. The results also show that MASE of all estimates of the shape parameter is increasing for an increase of the parameter value with all sample sizes
7. The results showed that the values of all MASE decrease as *n* increases

## 7. Conclusion

In this work, CLLF is developed to estimate the shape parameter of LD. The development occurred through merging the weights into the CLLF to generate a new loss function called the weighted compound LINEX loss function (WCLLF). We used WCLLF to estimate the LD shape parameter, through Bayesian and expected Bayesian estimation. Subsequently, six different types of loss functions are discussed, including SELF, LLF, ASLF, ENLF, and CLLF and the proposed loss function WCLLF. Then, Bayesian and expected Bayesian estimations are compared based on proposed loss function with the other methods, including MLE, Bayesian, and E-Bayesian estimators under different loss functions. The simulation results show that the Bayes estimator according to WCLLF and the E-Bayesian estimator according to WCLLF proposed in this work have the best performance in estimating the shape parameters based on the least mean averaged squared error. E-Bayesian estimators perform better than the Bayesian estimator in terms of MASE, for all sample sizes *n* and all cases. The results of the simulation showed that the E-Bayesian estimation method is both efficient and easy to perform.

## Figures and Tables

**Table 1:** The estimates for different Bayesian estimates of the parameter *β*.

*β*	*n*	${\hat{\beta }}_{\text{MLE}}$	${\hat{\beta }}_{\text{BSE}}$	${\hat{\beta }}_{\text{BSA}}$	${\hat{\beta }}_{\text{BEN}}$	${\hat{\beta }}_{\text{BL}}$		${\hat{\beta }}_{\text{BCL}}$		${\hat{\beta }}_{\text{BWCL}}$	
						*c*=0.5	*c*=1.5	*c*=0.5	*c*=1.5	*c*=0.5	*c*=1.5
1	25	1.044	1.046	1.026	1.006	1.035	1.026	1.047	1.046	1.023	1.025
	50	1.022	1.023	1.013	1.003	1.018	1.013	1.023	1.023	1.013	1.012
	75	1.012	1.013	1.006	0.910	1.001	1.008	1.013	1.014	1.006	1.007
	100	1.001	1.011	1.006	1.001	1.008	1.007	1.011	1.011	1.006	1.006

1.5	25	1.567	1.553	1.523	1.493	1.506	1.504	1.556	1.548	1.508	1.501
	50	1.527	1.522	1.507	1.492	1.499	1.503	1.522	1.524	1.499	1.501
	75	1.519	1.516	1.506	1.496	1.501	1.502	1.516	1.516	1.501	1.501
	100	1.514	1.511	1.504	1.496	1.500	1.504	1.511	1.515	1.500	1.503

2	25	2.088	2.049	2.008	1.969	2.007	1.975	2.050	2.052	1.968	1.969
	50	2.038	2.021	2.001	1.981	2.000	1.985	2.021	2.023	1.981	1.982
	75	2.031	2.020	2.006	1.993	2.006	1.992	2.020	2.016	1.993	1.989
	100	2.017	2.009	1.999	1.989	1.999	1.990	2.009	2.009	1.989	1.989

**Table 2:** The estimates for different E-Bayesian estimates of the parameter *β*.

*β*	*n*	${\hat{\beta }}_{\text{EBSE}}$	${\hat{\beta }}_{\text{EBSA}}$	${\hat{\beta }}_{\text{EBEN}}$	${\hat{\beta }}_{\text{EBL}}$		${\hat{\beta }}_{\text{EBCL}}$		${\hat{\beta }}_{\text{EBWCL}}$	
					*c*=0.5	*c*=1.5	*c*=0.5	*c*=1.5	*c*=0.5	*c*=1.5
1	25	1.043	1.022	1.002	1.032	1.022	1.043	1.042	1.021	1.021
	50	1.021	1.011	1.001	1.016	1.011	1.021	1.021	1.011	1.010
	75	1.012	1.005	0.998	1.008	1.006	1.012	1.013	1.005	1.006
	100	1.010	1.005	1.000	1.007	1.006	1.010	1.010	1.005	1.005

1.5	25	1.548	1.517	1.487	1.501	1.499	1.550	1.542	1.502	1.495
	50	1.519	1.504	1.489	1.496	1.500	1.519	1.521	1.496	1.498
	75	1.514	1.504	1.494	1.499	1.500	1.514	1.514	1.499	1.499
	100	1.510	1.502	1.495	1.498	1.503	1.510	1.513	1.498	1.502

2	25	2.042	2.001	1.962	2.001	1.968	2.043	2.045	1.961	1.963
	50	2.017	1.997	1.977	1.997	1.982	2.017	2.019	1.977	1.978
	75	2.017	2.004	1.990	2.003	1.989	2.017	2.014	1.990	1.987
	100	2.007	1.997	1.987	1.997	1.989	2.007	2.007	1.987	1.987

**Table 3:** MASE for different Bayesian estimates of the parameter *β*.

*β*	*n*	${\hat{\beta }}_{\text{MLE}}$	${\hat{\beta }}_{\text{BSE}}$	${\hat{\beta }}_{\text{BSA}}$	${\hat{\beta }}_{\text{BEN}}$	${\hat{\beta }}_{\text{BL}}$		${\hat{\beta }}_{\text{BCL}}$		${\hat{\beta }}_{\text{BWCL}}$	
						*c*=0.5	*c*=1.5	*c*=0.5	*c*=1.5	*c*=0.5	*c*=1.5
1	25	0.0503	0.0485	0.0452	0.0428	0.0456	0.0420	0.0485	0.0470	0.0431	0.0417
	50	0.0219	0.0216	0.0208	0.0202	0.0209	0.0203	0.0216	0.0214	0.0203	0.0202
	75	0.0139	0.0138	0.0134	0.0132	0.0135	0.0132	0.0138	0.0137	0.0133	0.0132
	100	0.0104	0.0103	0.0101	0.0100	0.0102	0.0101	0.0103	0.0104	0.0100	0.0101

1.5	25	0.1110	0.1005	0.0943	0.0902	0.0859	0.0841	0.1016	0.0971	0.0865	0.0834
	50	0.0498	0.0476	0.0463	0.0454	0.0444	0.0434	0.0478	0.0465	0.0444	0.0432
	75	0.0309	0.0300	0.0294	0.0290	0.0286	0.0299	0.0301	0.0313	0.0286	0.0298
	100	0.0238	0.0233	0.0230	0.0228	0.0225	0.0218	0.0234	0.0226	0.0225	0.0217

2	25	0.1952	0.1672	0.1584	0.1532	0.1516	0.1450	0.1679	0.1724	0.1408	0.1442
	50	0.0883	0.0822	0.0801	0.0789	0.0784	0.0745	0.0822	0.0807	0.0757	0.0742
	75	0.0565	0.0538	0.0527	0.0521	0.0520	0.0516	0.0538	0.0544	0.0507	0.0514
	100	0.0414	0.0400	0.0395	0.0393	0.0391	0.0384	0.0400	0.0398	0.0385	0.0383

**Table 4:** MASE for different E-Bayesian estimates of the parameter *β*.

*β*	*n*	${\hat{\beta }}_{\text{EBSE}}$	${\hat{\beta }}_{\text{EBSA}}$	${\hat{\beta }}_{\text{EBEN}}$	${\hat{\beta }}_{\text{EBL}}$		${\hat{\beta }}_{\text{EBCL}}$		${\hat{\beta }}_{\text{EBWCL}}$	
					*c*=0.5	*c*=1.5	*c*=0.5	*c*=1.5	*c*=0.5	*c*=1.5
1	25	0.0478	0.0447	0.0425	0.0450	0.0415	0.0479	0.0463	0.0426	0.0412
	50	0.0214	0.0207	0.0201	0.0208	0.0202	0.0214	0.0213	0.0202	0.0201
	75	0.0137	0.0134	0.0132	0.0134	0.0132	0.0137	0.0136	0.0132	0.0132
	100	0.0103	0.0101	0.0100	0.0101	0.0101	0.0103	0.0104	0.0100	0.0101

1.5	25	0.0994	0.0936	0.0898	0.0854	0.0836	0.1005	0.0961	0.0860	0.0829
	50	0.0474	0.0461	0.0453	0.0442	0.0433	0.0475	0.0463	0.0443	0.0431
	75	0.0299	0.0293	0.0290	0.0285	0.0298	0.0299	0.0312	0.0285	0.0297
	100	0.0233	0.0229	0.0227	0.0225	0.0217	0.0233	0.0225	0.0225	0.0216

2	25	0.1659	0.1577	0.1530	0.1509	0.1448	0.1667	0.1711	0.1407	0.1440
	50	0.0818	0.0799	0.0788	0.0782	0.0744	0.0818	0.0803	0.0757	0.0741
	75	0.0536	0.0526	0.0520	0.0519	0.0515	0.0536	0.0542	0.0506	0.0513
	100	0.0399	0.0395	0.0393	0.0391	0.0384	0.0399	0.0397	0.0385	0.0383

## Data Availability

The data used to support the findings of the study were generated by simulation done by using mathematical software.
